# Decreased respiratory symptoms in cannabis users who vaporize

**DOI:** 10.1186/1477-7517-4-11

**Published:** 2007-04-16

**Authors:** Mitch Earleywine, Sara Smucker Barnwell

**Affiliations:** 1 Department of Psychology, University at Albany, State University of New York, 1400 Washington Ave., SS369, Albany, New York, 12222, USA; 2 Department of Psychology, University of Southern California, SGM 501, Los Angeles, California, 90089-1061, USA

## Abstract

Cannabis smoking can create respiratory problems. Vaporizers heat cannabis to release active cannabinoids, but remain cool enough to avoid the smoke and toxins associated with combustion. Vaporized cannabis should create fewer respiratory symptoms than smoked cannabis. We examined self-reported respiratory symptoms in participants who ranged in cigarette and cannabis use. Data from a large Internet sample revealed that the use of a vaporizer predicted fewer respiratory symptoms even when age, sex, cigarette smoking, and amount of cannabis used were taken into account. Age, sex, cigarettes, and amount of cannabis also had significant effects. The number of cigarettes smoked and amount of cannabis used interacted to create worse respiratory problems. A significant interaction revealed that the impact of a vaporizer was larger as the amount of cannabis used increased. These data suggest that the safety of cannabis can increase with the use of a vaporizer. Regular users of joints, blunts, pipes, and water pipes might decrease respiratory symptoms by switching to a vaporizer

## Background

Cannabis smoke contains gaseous and particulate matter with the potential to create symptoms of respiratory problems [1]. Although cannabis creates fewer problems than cigarette smoking [2], increasing its safety has the potential to improve quality of life. One step toward increasing the safety of cannabis involves the use of vaporizers. Vaporizers heat cannabis to temperatures that release cannabinoids in a fine mist without creating the toxins associated with combustion [3,4]. Although vaporizers are not common knowledge in popular culture, a recent photograph of one appeared in the New England Journal of Medicine [5], and information about the machine is becoming more available. A vaporizer has the potential to increase the safety of cannabis use, but data from human users appear only rarely [4].

The potential for cannabis-induced lung problems is particularly important in light of frequent concurrent tobacco smoking. Cannabis use may prove especially detrimental in the production of respiratory symptoms in cigarette smokers. For example, one study revealed increased respiratory symptoms in cannabis dependent 21-year-olds, but particularly in those who also smoked cigarettes. Cannabis dependence in the absence of cigarette use led to symptoms comparable to smoking 1–10 cigarettes per day, but quickly escalated when cannabis and tobacco were combined [6].

## Method

We sought to identify the impact of vaporizers on respiratory symptoms. In an effort to target frequent cannabis users, three organizations committed to reforming drug laws were asked to send a query to their mailing lists for participation in a survey. Participants responded to an email request and had a chance to win a cash prize. Approximately 9,000 people replied, but we focused on those who had used cannabis at least once in the previous month. (More details of the data collection appear in a paper addressing other aspects of this sample [7].) In an effort to minimize the impact of other sources of respiratory symptoms, only those respondents who did not have cystic fibrosis or asthma and had never inhaled other drugs (inhalants, heroin, methamphetamine, or crack cocaine) were included. Those who reported that their primary method of administration of cannabis was oral ingestion were also omitted, because eating the plant should have no smoke-inhalation-induced respiratory effects.

### Participants

The 6,883 people who qualified included 4,493 men (65.3%) and 2,390 women. Ages ranged from 18 to 88 (Mean = 31.3, SD = 12.4). Education ranged from some high school to advanced degrees, with a median of some college but not a degree. Median income was $20,000 to $40,000 per year. Respondents were primarily Caucasian (87%) but included African Americans (1%), Native Americans (3%), Asians (3%) Latinos (4%), and people of mixed race (2%). Participants reported that their first cannabis use was at a mean age of 16.7 (SD = 4.2).

### Measures

#### Respiratory symptoms

Participants reported respiratory symptoms by answering six questions: Do you usually have a cough? Does your chest sound wheezy or whistling other than from colds? Are you troubled by shortness of breath when hurrying on the level ground or walking up a slight hill? Do you have to walk slower than most people your own age on the level ground because of breathlessness? Do you cough up phlegm in the morning? and Do you wake up at night with tightness in your chest? These questions revealed respiratory problems in cannabis-dependent individuals in previous work [6]. Symptoms were not particularly common (mean of the total symptom count = 0.80, SD = 1.1), but ranged from 0 to 6.

The sum of these items had significant skew that would preclude the use of parametric statistics, so we created two groups of participants: those who did (N = 3,016) and did not (N = 3,867) report respiratory problems. This dichotomized outcome served as the dependent variable.

#### Vaporizer use

Participants reported the technique they used most frequently when ingesting marijuana, and chose from blunts, joints, water pipes, pipes, edibles, vaporizers, and other. Only 152 participants (2.2%) reported vaporizing as their primary method for cannabis use.

#### Marijuana use

Although assessing the frequency of marijuana use has proven comparable to assessing the frequency of use for other drugs, assessing the quantity of consumption remains quite difficult. Standard units comparable to those found with alcohol or cigarettes are unavailable. Because respiratory effects of marijuana should covary with the amount used rather than the simple frequency of use, we asked participants to estimate the amount of marijuana they consumed per week in 1-gram joint equivalents. This rough estimate is necessarily imperfect, but has proven useful in previous work [8]. Participants reported amount of cannabis use in one-gram joint equivalents, which averaged 9.4 grams per week (SD = 11.9).

#### Cigarettes

Those who smoked cigarettes (4,829) began at a mean age of 16.0 (SD = 3.4). Cigarette smoking was generally light. Mean cigarettes per day was 8.6 (SD = 10.7) but ranged as high as 4 packs per day.

## Results

A simple chi-square test revealed that vaporizer users were less likely to report respiratory problems than participants who did not vaporize, with 100 of 152 vaporizer users (65.8%) reporting no respiratory problems, compared to 3767 of 6731 (56.0%), chi-square (1) = 5.8, p < .05. This analysis provided a rough look at the potential for vaporizers, and suggested that the machines could improve respiratory symptoms. Nevertheless, this analytic approach did not account for important covariates or address potential interactions, so we used logistic regression. We computed interactions by centering the variables to correct for non-essential multicollinearity and then multiplying [9]. We report the full model with all two-way interactions and the three-way interaction present, but deleting any of these effects did not change the pattern of results. A logistic regression analysis with age and sex as covariates revealed main effects for cigarettes, cannabis use, and vaporizer use. The interaction of cigarettes and marijuana was significant, as revealed in previous work [6]. In addition the interaction of marijuana use and vaporizer use was significant, all p-values < .05. (See Table 1.)

**Table 1:** Predicting Respiratory Symptoms (N = 6,883)

**Predictor**	**B**	**Standard Error**	**Wald Test**	**Odds Ratio**
Age	-0.013	.003	19.2*	0.98
Sex	0.351	.066	28.5*	1.42
Cigarettes	0.069	.004	371.9*	1.07
Marijuana	0.021	.003	42.9*	1.02
Vaporizer	-0.911	.424	4.6*	0.40
Cigarettes × Marijuana	-0.158	.035	20.4*	0.85
Vaporizer × Cigarettes	-0.025	.035	0.5	0.98
Vaporizer × Marijuana	0.072	.035	4.2*	1.07
Vaporizer × Cigarettes × Marijuana	-0.003	.003	0.8	0.99

* p < .05

## Discussion

These results suggest that the respiratory effects of cannabis can decrease with the use of a vaporizer. The data reveal that respiratory symptoms like cough, phlegm, and tightness in the chest increase with cigarette use and cannabis use, but are less severe among users of a vaporizer. Because a sample this large can produce statistically significant effects that might not be clinically meaningful, a focus on odds ratios could prove fruitful. The odds ratio suggests that vaporizer users are only 40% as likely to report respiratory symptoms as users who do not vaporize, even when age, sex, cigarette use, and amount of cannabis consumed are controlled. The use of cigarettes in conjunction with cannabis exacerbated symptoms, as found in previous work [6]. The interaction between vaporizer use and cannabis consumption also appeared, suggesting that a vaporizer should have more impact on respiratory symptoms in people who use more marijuana. Odds ratios suggest that these effects are relatively small, but interactions often prove difficult to detect at all [9].

Several important limitations of these data deserve mention, particularly those related to sampling, Internet reporting, limitations of our measures, and the lack of random assignment to vaporizer use. In an effort to find regular users of cannabis, we targeted people with a potential interest in changing cannabis policy. These individuals might have consciously or unconsciously minimized their reports of symptoms that might cast cannabis in a negative light. Nevertheless, literally thousands of participants admitted to experiencing respiratory symptoms. The symptoms covaried with cannabis use, cigarette use, and the interaction of the two, as work with samples gathered in other ways has revealed [6]. These results suggest that reports among these participants are comparable to those found in other work. Any bias in reporting remains a problem, and only further work can help address this issue.

Vaporizer users might be more inclined to minimize respiratory symptoms than people who smoke cannabis in other ways. The price of a vaporizer can range as high as hundreds of dollars. Vaporizers also lack some of the convenience of other methods of marijuana use. Users who have spent this much money and effort might minimize reports of their respiratory symptoms, consciously or inadvertently, in an effort to justify their actions. Only a more objective measure of respiratory function that does not rely on self-report can sidestep this potential problem. Laboratory measures of lung function would make a nice addition to further work on this topic. Nevertheless, roughly 1/3 of the participants who used a vaporizer (52/152) did report symptoms, suggesting that self-report biases on symptom reports likely does not account for the entire phenomenon.

The use of the Internet for this type of work has advantages and disadvantages as well. This approach might lead individuals who are unwilling to travel to the laboratory to participate, potentially increasing generalizability. Heavy users with severe symptoms might be particularly disinclined to participate without the convenience of the Internet.

Recent work also suggests that people report more drug use while using the Internet than they do on standard paper-and-pencil measures [10]. Nevertheless, because Internet access was required for participation, these data might not generalize to meaningful subsets of the population without such access.

Our measures of vaporizer use and respiratory symptoms could also have been more detailed. A single question about the primary technique used for administering cannabis neglects potentially meaningful variation in vaporizer use. Some participants might use a vaporizer primarily but also smoke cannabis. In contrast, other participants might use a vaporizer exclusively. Both of these groups of participants would end up in the group who uses a vaporizer primarily. Vaporizers come in several forms, including conduction-style machines that employ a hot plate as well as convection-style devices that use warmed air. The efficacy of these different machines could vary substantially, but we could not address the question with the current data. These limitations, however, should decrease power rather than create a spurious result. By lumping participants who occasionally smoke cannabis into the same group with those who vaporize exclusively, we actually weaken the ability to detect effects. Including any type of vaporizer, no matter how effective, also has the potential to weaken effects. In a sense, the current study's estimate of the effect of a vaporizer on respiratory symptoms might be an underestimate of the improvement that could arise from a good vaporizer used as the exclusive method for ingesting cannabis. We also did not assess the length of time each participant had used a vaporizer. A vaporizer's impact might grow more dramatic with longer use. The assessment of respiratory symptoms was also not particularly elaborate, but the same measures revealed a significant impact of cannabis and cigarettes in this sample and in other work [6].

Finally, the use of a vaporizer was not randomly assigned. The possibility exists that cannabis users who choose a vaporizer might engage in a host of other behaviors designed to minimize respiratory symptoms, or simply be more health conscious in general. Like any correlational study, this one cannot address the role of causality. The current data are consistent, however, with the idea that cannabis vaporizers can decrease respiratory symptoms in regular users of the plant. A better test of a vaporizer's potential for minimizing problems would require recruiting cannabis smokers who report respiratory troubles, randomly assigning a group to use a vaporizer, and assessing any decrease in symptoms. The current data suggest that such an intervention could prove helpful.

Although the use of a vaporizer has the potential to increase the safety of cannabis as far as respiratory symptoms are concerned, pulmonary problems are not the only potential negative consequences of the plant. Reviews suggest that 9–12% of cannabis users develop symptoms of dependence [11]. Cannabis can lead to impaired driving skills [12], and heavy use in adolescence might create deviant brain structure [13] as well as decreases in intelligence [14]. A vaporizer offers no protection against these negative consequences. Nevertheless, a vaporizer has considerable potential for increasing cannabis drug safety by minimizing pulmonary troubles.

## Competing interests

ME is affiliated with organizations devoted to changing cannabis laws.

## Authors' contributions

ME contributed to study design, coordination and supervision, data analysis and interpretation, and drafted the manuscript. SSB participated in study design and coordination and helped draft the manuscript.
